# Fatal deep venous thrombosis and pulmonary embolism secondary to melioidosis in China: case report and literature review

**DOI:** 10.1186/s12879-019-4627-6

**Published:** 2019-11-21

**Authors:** Hua Wu, Dongliang Huang, Biao Wu, Mengjie Pan, Binghuai Lu

**Affiliations:** 1 Department of Laboratory Medicine, Hainan General Hospital, No 19 Xiuhua Street, Xiuying District, Haikou, 570311 China; 2 Department of Emergency, Hainan Armed Police Corps Hospital, Wenmingdong Road, Meilan District, Haikou, 570203 China; 3 Department of Infectious Diseases, Hainan General Hospital, No 19 Xiuhua Street, Xiuying District, Haikou, 570311 China; 4 Department of Radiology, Hainan General Hospital, No 19 Xiuhua Street, Xiuying District, Haikou, 570311 China; 5 Laboratory of Clinical Microbiology and Infectious Diseases, Department of Pulmonary and Critical Care Medicine, China-Japan Friendship Hospital, No 2, East Yinghua Road, Chaoyang District, Beijing, 100029 China; 6 Center for Respiratory Diseases, China-Japan Friendship Hospital, No 2, East Yinghua Road, Chaoyang District, Beijing, 100029 China; 7 National Clinical Research Center of Respiratory Diseases, No 2, East Yinghua Road, Chaoyang District, Beijing, 100029 China

**Keywords:** *Burkholderia pseudomallei*, Melioidosis, Deep venous thrombosis, Pulmonary embolism

## Abstract

**Background:**

*Burkholderia pseudomallei* is a gram-negative bacterium and the causative pathogen of melioidosis, which manifests a variety ranges of infection symptoms. However, deep venous thrombosis (DVT) and pulmonary embolism (PE) secondary to bacteremic melioidosis are rarely documented in the literature. Herein, we reported a fatal case of melioidosis combined with DVT and PE.

**Case presentation:**

A 54-year-old male construction worker and farmer with a history of diabetes was febrile, painful in left thigh, swelling in left lower limb, with chest tightness and shortness of breath for 4 days. He was later diagnosed as DVT of left lower extremity and PE. The culture of his blood, sputum and bone marrow samples grew *B. pseudomallei*. The subject was administrated with antibiotics (levofloxacin, cefoperazone/tazobactam, and imipenem) according to antimicrobial susceptibility testing and low molecular heparin for venous thrombosis. However, even after appropriate treatment, the patient deteriorated rapidly, and died 2 weeks after admission.

**Conclusions:**

This study enhanced awareness of the risk of *B. pseudomallei* bloodstream infection in those with diabetes. If a patient has predisposing factors of melioidosis, when DVT is suspected, active investigation and multiple therapeutic interventions should be implemented immediately to reduce mortality rate.

## Background

*Burkholderia pseudomallei*, a gram-negative bacterium commonly found in soil and water, is the infective pathogen of melioidosis in southeast Asia and northern Australia [1, 2]. Southern China has been in the expanded endemic zone of melioidosis [3–5]. Melioidosis involves almost any part of the body, with its clinical manifestation ranging from acute septicemia, respiratory tract infection, to chronic cutaneous infection. It is also called the great mimicker, as its pathogenesis and clinical features might present like tuberculosis or brucellosis [6, 7].

Furthermore, there are some predisposing risk factors for melioidosis, including smoking, diabetes, occupation, and history of exposure to contaminated soil or water prior to their illness [3, 8, 9]. Deep venous thrombosis (DVT) and pulmonary embolism (PE) caused by *B. pseudomallei* will reinforce the high mortality rate associated with this infection, but rarely documented in the literature [10, 11]. Herein, we reported a fetal case of DVT and PE secondary to bacteremic melioidosis in a diabetic farmer in mainland China alongside a literature review of *B. pseudomallei*-caused venous thrombosis.

## Case presentation

### Medical history

**Fig. 1:**
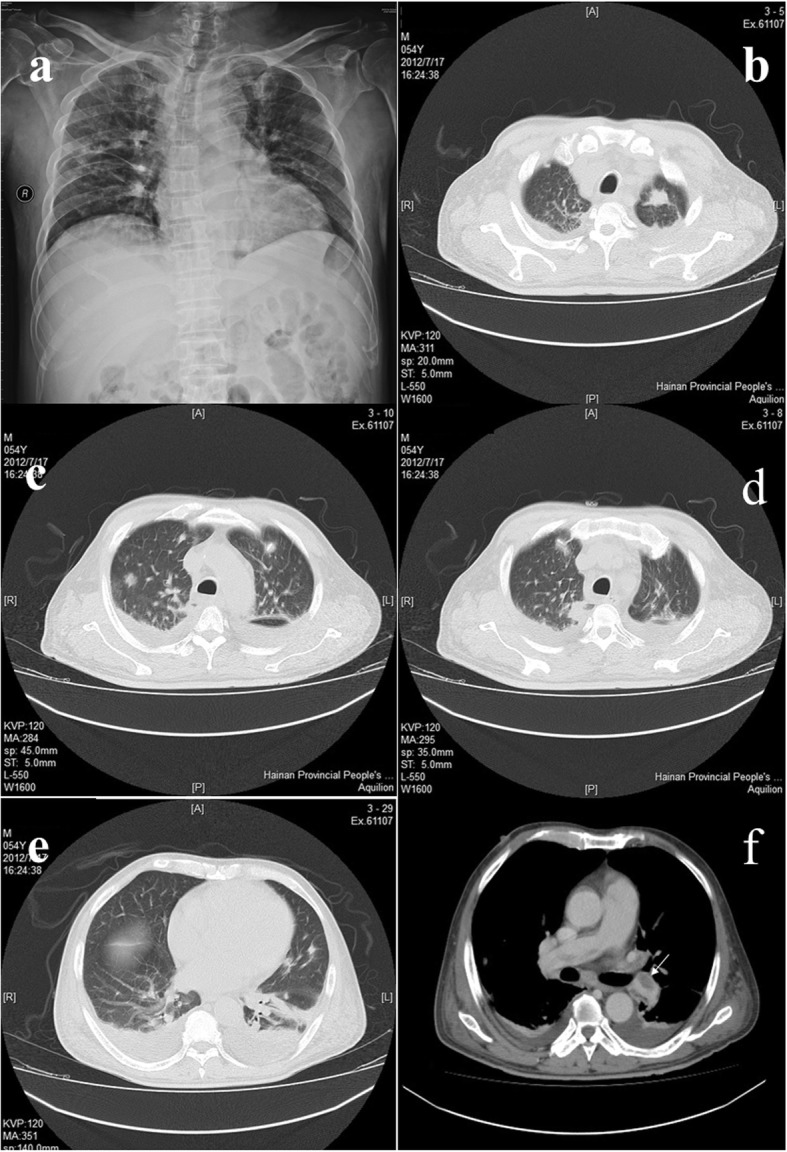
An X-ray image **a**: flaky blurred shadows with multiple small patches distributed in bilateral lungs with uneven density and ill-defined boundary; **b-e** pulmonary CT images: the enhanced irregular nodule in the left upper, and multiple point- and strip-like lesions scattered in the lower lobes. **f** contrast-enhanced CT: strip-like filling defect in the bilateral pulmonary arteries and their branches. Furthermore, the bilateral pleural effusion was revealed and no abnormality was discovered in the structure of the chest wall

A 54-year-old male construction worker and farmer, a resident in Sanya city, Hainan province, was admitted to Hainan General Hospital on July 14, 2012, and complained of chills, fever, cough, chest tightness, pain in left thigh and popliteal fossa, and left lower limb swelling for 4 days. Ultrasonography showed a slightly stronger echo in his left common femoral vein, revealing venous thrombosis. Imaging findings of X-ray on July 14 showed that flaky blurred shadows with multiple small patches scattered in bilateral lungs, with uneven density and ill-defined boundary (Fig. 1a). Furthermore, computed tomography (CT) on July 17 showed the mildly-enhanced irregular nodule with the size of 1.5 ✕ 1.7 cm in the left upper. The multiple point- and strip-like lesions scattered in the lower lobes (Fig. 1b-e). The contrast-enhanced CT scan demonstrated strip-like filling defect in the left and right pulmonary arteries and their branches (Fig. 1f). The bilateral pleural effusion was revealed and no abnormality was discovered in the structure of the chest wall. Moreover, in Fig. 1a, chest X-ray revealed multiple nodular opacities in bilateral lungs (including retrocardiac area), in Fig. 1b, c, and e, chest CT revealed multiple nodular opacities in bilateral lungs, and in Fig. 1d showed a peripheral wedge-shape opacity in right upper lobe. Taken together, these hinted the occurrence of septic PE in our patient. There were slightly larger mediastinal lymph nodes, bilateral pleural effusion, low-density lesions in the right lower kidney, and splenomegaly with multiple low-density lesions. His plasma D-dimers were 8.24 μg/ml (normal range≦0.5 μg/ml). Therefore, the diagnosis of DVT at left lower extremity and PE was made. At admission, the patient’s body temperature rose up to 39 °C, and his blood examinations, including leucocyte, neutrophil percentage and C-reactive protein, elevated significantly over reference ranges. He was suspected of severe infection, and levofloxacin and cephalosporin were empirically administrated the day after his hospitalization. The patient refused the implantation of inferior vena cava filter. After July 17, he was treated with daily subcutaneous injections of low molecular weight heparin to reduce the risk of further thrombosis. Despite anticoagulation and antimicrobial therapy, the patient presented continuous high fever, chest tightness, cough and expectoration, shortness of breath, and antibiotic was transferred to cefoperazone/tazobactam.

His coagulation results were normal except for the elevated fibrinogen level (4.17 g/L, normal rang 2~4 g/L). Blood examination demonstrated hyperglycemia (blood glucose7.98 mmol/L, normal range 3.89~6.11 mmol/L) and hyperlipemia (triglyceride 2.59 mmol/L, normal range 0.33~1.69 mmol/L), but antibody examinations for human immune deficiency virus, hepatitis B virus and hepatitis C virus were all negative. Expectorated sputum smear revealed a large amount of polymorphonuclear leucocytes and gram-negative bacilli. On July18 to 22, his blood, sputum and bone marrow grew *B. pseudomallei*, respectively. Antimicrobial susceptibility testing (AST) showed that the strain was susceptible to all antimicrobial agents with breakpoint value in CLSI.

On July 20, the patient began receiving antimicrobial therapy of imipenem. However, 4 days later, his condition dramatically deteriorated, and presented as heart failure, renal failure and persistent high fever (up to 40.1 °C). On July 25, his family members received the medical crisis notice of life-threatening respiratory and circulatory failure. Considering the low probability of his survival, his family decided to take him home in accordance to the local customs of not dying in hospital. Without proper treatment the patient died 3 days later after he was discharged.

### Microbiologic test

**Fig. 2:**
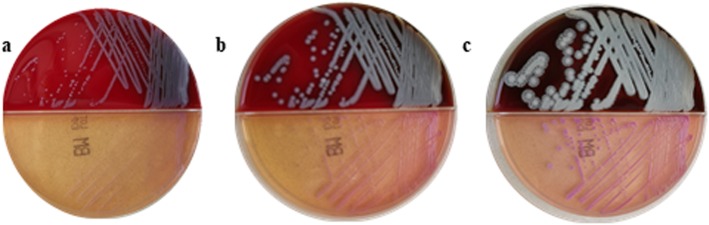
**a**-**c** *B. pseudomallei* colonies after 24 h (**a**), 48 h (**b**) and 72 (**c**) of incubation on blood and MacConkey agar, respectively

After 24 h of incubation on blood agar at 37 °C in a 5% CO_2_ atmosphere, the *B. pseudomallei* B86 grew into round, wet, convex, non-hemolytic, gray-white colonies, with1mm in size. But after another 24 h, the colonies turned dry, flat, a little hemolytic, and yellow with a little metallic luster, and with the smell of earthy mildew. After 72 h, the hemolytic zone, metallic luster and smell became stronger, colonies wrinkled as wheel-shaped (Fig. 2a-c). Phenotypic identification by DL-96NE (Zhuhai DL biotech, China) revealed *B. pseudomallei*, and the identification rate was 99.5%. AST was performed using E-test method (Liofilchem, Italy), the MIC results were as follows: susceptible to imipenem (MIC: 0.5 μg/ml), ceftazidim (MIC: 1 μg/ml), amoxicillin/clavulanate (MIC: 2 μg/ml), doxycycline (MIC: 2 μg/ml), and trimethoprim/sulfamethoxazole (MIC: 2 μg/ml).

### Identification by 16S rRNA sequencing and multilocus sequence typing (MLST) of BP86

To genetically characterize the isolate, the 16S rRNA sequencing was conducted. Sequence analysis of the 1385 bp-segment of 16S rRNA gene of BP86 demonstrated an identity of 99.93% with *B. pseudomallei* K96243 (GenBank accession no. NC_006351.1). The whole genome of the pathogen was also sequenced using a whole-genome shotgun strategy based on the Illumina HiSeq platform. The selected optimal assembly results were compared with the seven housekeeping genes of *B. pseudomallei* for MLST by reference to https://pubmlst.org/bpseudomallei/, and the determined sequence type of BP86 was ST 46.

## Discussion and conclusions

*B. pseudomallei* is the pathogen of melioidosis, an infectious disease involving almost every system all over the body with complicated clinical characteristics. Melioidosis is generally thought to be epidemic in tropical and sub-tropical zone, but recently, it is speculated that it is distributed more widely beyond the tropics based on increased case reports and predictive modelling studies [12]. Southern China, especially Hainan province, is one of the additional endemic areas [1, 5]. As reported by Zheng X et al., there are approximately 20 to 30 culture-confirmed melioidosis cases in Hainan General Hospital [5]. Between 2002 and 2013, in another research in Hainan province, not including the cases in Hainan General Hospital, 170 cases of melioidosis were documented, and the most common presentations were pneumonia and bacteremia [4].

*B. pseudomallei* is found in soil and stagnant water in endemic regions, and it usually invades the epithelial cells of the mucosal surface or skin and then spread to others [12–16]. Diabetes mellitus is the most common predisposing factor of melioidosis, and more than 50% of the world’s melioidosis patients are diabetic [8, 9]. In the present study, our patient was a diabetic construction worker and farmer living in the endemic area, and therefore, the most possible way of being infected might be via percutaneous inoculation or inhalation of contaminated soil or water in the environment [14].

Melioidosis has multiple clinical manifestations, including acute severe sepsis, septic shock, infectious multiple organ failure, and skin and soft tissue infections. In addition to the symptoms of common bacterial infections, melioidosis is often misdiagnosed as tuberculosis or quite similar to cancer, thus it is called the remarkable imitator [3]. Few thrombosis cases caused by melioidosis were reported. A review of the medical literature was performed through PubMed using the following combination of MeSH terms: (melioidosis OR *Burkholderia pseudomallei* OR thrombosis) (https://www.ncbi.nlm.nih.gov/pubmed), only six cultured-confirmed cases of melioidosis thrombosis could be found, and three of which were dural venous sinus thrombosis [10, 17, 18], two cases were splenic vein thrombosis [19, 20], and one was portal vein thrombosis [11]. Characteristics of the melioidosis with venous thrombosis, including predisposing factors, clinical presentation, treatment and outcome are detailed in Table 1. Inflammation has been regarded as a common pathway through which various risk factors trigger vein thrombosis. The sepsis could release inflammatory mediators, cause endothelial injury, and stimulate the production of plasminogen activator inhibitor-1 (PAI-1), resulting in abnormal function of coagulation-anticoagulation-fibrinolysis system [21, 22]. For melioidosis sepsis in particular, inflammatory response might result in the reduction of the endothelial modulators protein C and antithrombin [10, 23]. In line with the study by Hernández-Espinosa D et al., diabetes is not just the most common risk factor of melioidosis, but also might activate the activity of protein kinase C and aldose reductase, and lead to thrombosis [24]. Our subject was a diabetic patient with a badly-controlled blood glucose level. Furthermore, according to our review results, three out of six (50%) patients with vein thrombosis were diabetic. In line with previous studies [14, 20, 25], a male preponderance was documented in melioidosis. All melioidosis patients with thrombosis in our reviewed cases were male.

**Table 1:** Literature review of melioidosis thrombosis cases with summary of predisposing factors, clinical presentation, treatment and outcome

Reference	2006 [10]	2007 [16]	2018 [17]	2010 [18]	2015 [19]	2017 [11]	Our data
Cases number	1	1	1	1	1	1	1
Country/Area	Thailand	Malaysia	India	Brunei	France	Thailand	China
Age(y)/Gende	42/male	33/male	23/male	48/male	52/male	54/male	54/male
Underlying disease	diabetes, mild alcoholic cirrhosis	NA	NA	diabetes	NA	diabetes	diabetes
Presentation	fever, headache, left hemiparesis, focal seizure, increased intracranial pressure	fever, seizures, altered conscious level and neck stiffness,	diplopia, decreased hearing	fever, rigor, abdominal pain, loss of appetite, weight loss	fever with chills, palpitations and an unproductive cough.	febrile, icteric, dehydrated	febrile, painful in left thigh with left lower limb swelling, chest tightness, shortness of breath
Thrombus site	dural sinus	superior sagittal sinus	dural venous sinus	splenic vein	splenic vein, right portion of the portal vein	left portal vein	left lower extremity
Sources Identification	blood	blood	brain tissue biopsy	blood, pancreas, lung, spleen, liver	blood, sputum	blood	blood, sputum, bone marrow
Antibiotic therapy	ceftazidime	NA	ceftazidime, cotrimoxazole	ceftazidime, amoxicillin-clavulanic acid, doxycycline, co-trimoxazole	ceftazidime, meropenem, co-trimoxazole, amoxicillin-clavulanic acid,	ceftazidime, amoxicillin-clavulanic acid	levofloxacin, cefoperazone/tazobactam, imipenem
Other therapy	antiepileptic drug and anticoagulant	craniectomy and drainage of the abscess	anticoagulant	no other therapy	anticoagulant	anticoagulant	anticoagulant (low molecular heparin)
Outcome	recovered	recovered	no improvement	recovered	recovered	recovered	died

^*^NA Not available

Five of six reported and our case *B. pseudomallei* strains were isolated from blood culture, and in an Indian young male, the microorganism was recovered from brain tissue biopsy [18]. Furthermore, in our case, bone marrow also grew *B. pseudomallei*. Melioidosis has a case-fatality rate as high as 40% [3, 25], and the prognosis may be worse if combined with DVT/PE. However, to our surprise, in line with our review, five cases in literature with DVT/PE recovered and discharged home and one remained unchanged. Unfortunately, our patien was complicated with PE, and died afterwards, even timely anticoagulation and susceptible antibiotics were used.

In summary, to the best of our knowledge, this is the first report of PE/DVT secondary to *B. pseudomalle*i infection. Physicians and laboratories in endemic areas should be aware of this potentially emerging disease. DVT/PE or other thrombosis should be taken into account when patients have predisposing factors or culture-confirmed melioidosis, especially if the patient has pain and swelling in the limb and chest pain.

## Data Availability

All the data and material involved in the current study are available from the corresponding author on reasonable request.

## Abbreviations

AST: Antimicrobial susceptibility testing
CT: Computed tomography
DVT: Deep venous thrombosis
MLST: Multilocus sequence typing
PE: Pulmonary embolism
